# Association Analysis of Arsenic-Induced Straighthead in Rice (*Oryza sativa* L.) Based on the Selected Population with a Modified Model

**DOI:** 10.1155/2017/7641362

**Published:** 2017-07-25

**Authors:** Xiaobai Li, Biaolin Hu, Xuhao Pan, Ning Zhang, Dianxing Wu

**Affiliations:** ^1^State Key Lab of Rice Biology, IAEA Collaborating Center, Zhejiang University, Hangzhou, Zhejiang, China; ^2^Zhejiang Academy of Agricultural Sciences, Hangzhou, China; ^3^Rice Research Institute, Jiangxi Academy of Agricultural Sciences and Nanchang National Sub-Center for Rice Improvement, Nanchang, Jiangxi, China; ^4^Institute of Tobacco Research of Chinese Academy of Agricultural Sciences, Qingzhou, Shandong, China

## Abstract

A rice physiological disorder makes mature panicle keep erect with empty grains termed as “straighthead.” Straighthead causes yield losses and is a serious threat to rice production worldwide. Here, a new study of association mapping was conducted to identify QTL involved in straighthead. A subset of 380 accessions was selected from the USDA rice core collection and genotyped with 72 genome-wide SSR markers. An optimal model implemented with principle components (PCs) was used in this association mapping. As a result, five markers were identified to be significantly associated with straighthead. Three of them, RM263, RM169, and RM224, were consistent with a previous study. Three markers, RM475, RM263, and RM19, had a resistant allele associated with a decrease in straighthead rating (straighthead rating ≤ 4.8). In contrast, the two other marker loci RM169 and RM224 had a few susceptible alleles associated with an increase in straighthead rating (straighthead rating ≥ 8.7). Interestingly, RM475 is close to QTL “*qSH-2*” and “*AsS*” with straighthead resistance, which was reported in two studies on linkage mapping of straighthead. This finding adds to previous work and is useful for further genetic study of straighthead.

## 1. Introduction

A physiological disorder of rice (*Oryza sativa* L.) exhibits a typical symptom with sterile florets and distorted palea and lemma [1]. The panicles bear unfilled grains and stay upright, while empty hulls are often distorted into a crescent or parrot-beak shape. That is why people call it by “straighthead.” In an extreme case, yield losses can approach 100%. The first report of straighthead dates from 1912 in southern states of the United States (US), including Arkansas, Louisiana, and Texas [2]. Besides, straighthead has been reported in Portugal [3], Thailand [4], Japan [5], Australia [6], and Argentina [7]. Breeding resistant cultivars is an efficient strategy to overcome straighthead. Marker assistant selection (MAS) will accelerate the breeding process. Association mapping is promising for identifying causative polymorphisms for complex traits [8] because it takes advantage of accumulated historic recombination events in natural populations and can evaluate multiple alleles simultaneously [9, 10]. Diverse collections (population) have been extensively used for association mapping. However, the discrepancy exists in allele frequencies of many diverse populations and statistic models, which can dramatically influence the power for mapping the associated alleles [11]. Therefore, it is necessary to recheck the marker alleles associated with the QTL in detail. In this study, we optimized a model of association mapping and selected a subset of accessions with good phenotypic replication to map the genes responsible for straighthead in rice. The population for association mapping was developed from the USDA rice core collection as previously described [12, 13].

## 2. Materials and Methods

### 2.1. Plant Materials

A total of 990 accessions were selected from the USDA rice core collection [13] for straighthead evaluation based on maturity and plant status in a 2002 field test [12]. The field was treated with the arsenic containing herbicide monosodium methanearsonate (MSMA). MSMA can induce the symptom of straighthead and thus is commonly used for evaluating straighthead in rice. These accessions were planted in a randomized complete block design with three replications. As a control two straighthead resistant cultivars and two straighthead susceptible cultivars [1] were included in the center of each tier containing 99 plots. The resistant cultivars were Zhe733 and Jing185 and susceptible cultivars were “Cocodrie” and “Mars.” Straighthead was rated separately for early, intermediate, and late maturity groups of the accessions in August, September, and October, respectively. Because more than 80% of these accessions were highly susceptible to straighthead, 380 accessions were selected for association mapping.

### 2.2. Phenotyping and Genotyping

Straighthead was visually rated at maturity based on floret fertility or sterility and panicle emergence from the flag leaf sheath, with nine levels from resistance to susceptibility [1]. DNA was extracted and genotyped with one indel and 70 SSR markers covering the entire rice genome. Sixty-eight of these markers were obtained from http://www.gramene.org. The other two (AP5652-1 and AP5652-2) were developed from the BAC AP5652 in house as described by Li et al. [15]. PCR and electrophoresis were carried out according to the procedures [15].

### 2.3. Statistical Analysis

The model-based program* INSTRUCT* [16] was used to infer population structure using a burn-in of 50,000 runs, Markov chain Monte Carlo to 50,000 iterations, and a model allowing for admixture and correlated allele frequencies.* INSTRUCT* eliminates the assumption of Hardy–Weinberg equilibrium within clusters [16]. The population structure was graphically displayed using* Distribute* [17]. The number of groups (*K*) was set from 1 to 10, with 5 independent runs each, to identify the *K* with the highest log likelihood. The most probable structure number (*K*) was decided based on log probability-Ln*P*(*D*) and deviance information criteria (DIC). An accession was assigned to a single group with its inferred maximum ancestry (*Q*) from the group. To validate the genetic structure and test for different models, principal components analysis (PCA) was performed with* NTSYSpc *software version 2.11V [18]. The PCA was performed on the correlation matrix and summarizes the major patterns of variation in a multilocus data set. The first three principal components were used to visualize the dispersion of accessions. Genetic distance was calculated using Nei's genetic distance [19]. The best fit model was selected for mapping after model comparison. The model comparison were conducted as described by Li et al. [15, 20]. In order to reduce the risk of false marker-trait associations with high *P* inflation, a false-discovery rate (FDR) was calculated using MULTTEST procedure in SAS v9.2 [21]. Effects of allele at marker loci associated with straighthead were compared for least square means (LSMEANS option of PROC MIXED).

## 3. Results

In the collection, MSMA in the field induced straighthead with symptoms of distorted florets in the partially emerged panicles and no seed set at all for the susceptible check cultivars. As a result, the straighthead score in resistant check Zhe733 was categorized as “class I” and the susceptible checks Cocodrie and Mars were categorized as “class III.” Based on the straighthead score, the 380 accessions had 37 accessions classified into rating of 1–4, 168 into rating of 4.1–6.9, and 175 into rating of 7.0–9.0.

The set of 72 markers with a genome-wide distribution detected a total of 677 alleles in the collection of 380 accessions. The average number of alleles per locus was 9.54 ranging from 2 (RM507, RM338, RM455, and Rid12) to 30 (RM11229). Polymorphic information content (PIC) varied from 0.08 for AP5625-1 to 0.91 for RM11229 among these markers, with an average of 0.60.

**Figure 1:**
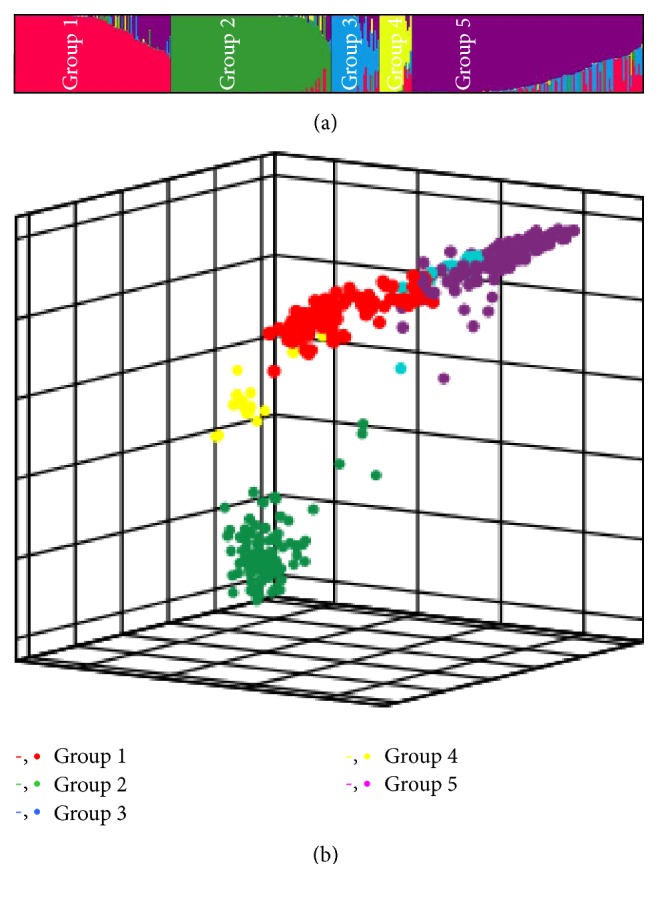
The genetic structure of 380 accessions for the analysis models of association mapping. (a) Estimated group structure is partitioned into five colored groups that represent the individual estimated levels of the five clusters and (b) principal component analysis (PCA) shows the accessions' pattern of spatial distribution. Each color represents one of the five groups indicated by the ancestry index. Each “-” or “•” represents one accession in (a) or (b), respectively.

**Figure 2:**
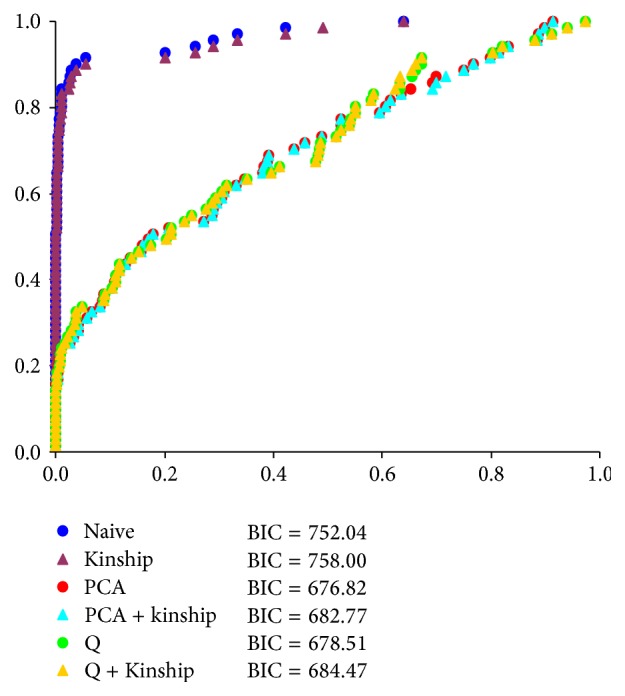
The selection of the best fit model for association mapping of straighthead. Comparative plots of observed* versus* expected *P* values and Bayesian information criterion (BIC) for six different association mapping models using 71 markers among 380 accessions. With an assumption that these markers are unlinked to the polymorphisms controlling straighthead, methods that appropriately control Type I error should show a uniform distribution of *P* values and have low BIC scores. The PCA model was selected as the best fit model for association mapping of straighthead due to the lowest BIC score and a uniform distribution of *P* values.

Five model-based groups were identified by* INSTRUCT *and accessions were assigned into their corresponding groups according to their Q (ancestry index) (Figure 1(a)). The first three components of PCA containing 56.36% variation among the 380 accessions also exhibited a similar pattern of genetic structure (Figure 1(b)). All these approaches concluded that the five-group structure sufficiently explained genetic variation in the collection. Relative performance assessed by BIC among six models (naive, kinship, PCA, PCA + kinship, Q, and Q + kinship) for straighthead is present in Figure 2. The five dimensions of PCA were determined based on BIC and applied in PCA and PCA + kinship models. The PCA model had the smallest BIC score among the six models; thus it was selected as the best fit model for association mapping of straighthead.

**Figure 3:**
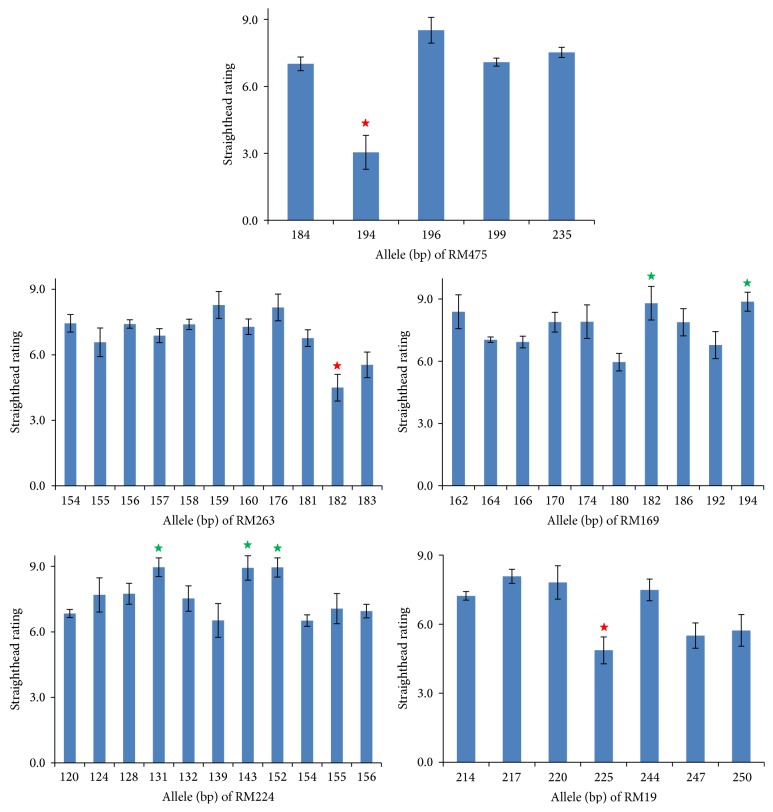
Deep analysis of the markers significantly associated with straighthead in terms of alleles. Alleles involved in straighthead resistance as shown by a decrease in effect of straighthead rating are indicated with a red star. Alleles involved in susceptibility of straighthead with an increased effect on straighthead rating are shown with a green star.

Using the selected model, five marker loci were identified to be significantly associated with straighthead at the 0.01% level of qFDR (Table 1). The allelic effect for each associated loci could be estimated with the mean phenotypic value using LSMEANS statement in PROC MIXED. Comparisons among alleles were tested with option PDIFF of LSMEANS statement individually for each marker. Allelic comparisons of the five marker loci displayed variation of their effects among alleles at the same locus (Figure 3). Three markers, RM475, RM263, and RM19, had a specific allele associated with a decrease in straighthead rating (straighthead  rating ≤ 4.8) compared to the other alleles at their respective loci. In contrast, the two other marker loci RM169 and RM224 had a few alleles associated with an increase in straighthead rating (straighthead  rating ≥ 8.7) compared to other alleles at the same locus. Three of the five markers were the same as those identified by Agrama and Yan [10]. However, RM475 with allele 194 bp, which had the largest effect (3.3) for straighthead resistance among all the alleles (Figure 3), had not been reported previously [10]. Interestingly, RM475 was also identified to link to straighthead in a cross population [14].

## 4. Discussion

### 4.1. Model Comparison and Association Mapping

The simulations of Type I error estimated by BIC and visual plots of the observed and expected *P* values for all models suggest three important points (Figure 2). First, the four models (Q, PCA, Q + kinship, and PCA + kinship) that account for population structure have fairly low BICs, which help control spurious associations and Type I error. Furthermore, naive and kinship models which do not account for population structure have very high BICs and an elevated risk of increased Type I errors. Second, kinship via ancestral relationship makes no improvement to the model as indicated by the highest BIC among the six models. PCA + kinship and Q + kinship models have higher BIC values than PCA and Q models, respectively. Shao et al. [22] has observed that the Q + kinship model performed similarly to the Q in their rice populations. The similar result has been observed in two another rice studies [15, 20]. However, kinship does improve the accuracy of association mapping results in studies with humans and cross pollinated crops [23, 24]. In our study, kinship among accessions may have less effect due to the low complexity of relatedness that results from the restricted gene flow of rice's self-reproductive mode and high genetic diversity represented in our rice panel [20, 25, 26]. Third, the PCA model with five dimensions performs better than the Q model based on five groups according to its smaller BIC. PCA is a fast and effective way to diagnose population structure [27, 28] and can handle a large number of markers and correct for subtle population stratification without being restricted by Hardy–Weinberg equilibrium [29–31]. These features of PCA may explain its increased performance over the Q model to control Type I error in our rice collection. Due to varying genetic composition, model selection should be used to determine the best model to use for association mapping with a specific population [15, 20].

Using the PCA model for our population, we identified five markers associated with straighthead (Table 1). Three of them, RM263, RM169, and RM224, were consistent with a previous study by Agrama and Yan [10] where seven markers were reported to associate with straighthead. This study identified an additional novel marker associated with straighthead resistance, RM475, which was confirmed by linkage mapping with the F_2_ population of Zhe733/R312 [14]. The 194 bp allele of RM475 was associated with the highest level of straighthead resistance as indicated by its strong effect (Figure 3). The reasons for different results between the present study and Agrama and Yan's [10] association mapping may be that (1) the distribution of allele frequencies and linkage disequilibrium (LD) may be substantially different between the two populations for mapping because they were built independently and consisted of different lines. Different allele frequencies and LD would result in different results of association mapping [32, 33]; and (2) in order to control the spurious association or Type I error, we compared six models based on the BIC method and identified the best fit model “PCA” to map with our data. Therefore, the present study improves the results of previous study through the use of more stringent testing standards. Additionally this study validates previous findings through the use of a new rice panel composed of different accessions compared to the previous study and confirms the results of the novel marker identified in the study through the use of conventional linkage mapping with a segregating population.

### 4.2. Comparison of QTLs in Association Mapping with the Previous Ones

Occasionally markers linked to specific QTL are not always identified in association mapping. For example, while RM44 near* qSH-8* on Chr 8 was included in the association study it did not yield a significant association.* qSH-8 *is a major straighthead QTL reported in two previous studies [14, 34]. This could be due to a low level of polymorphism of RM44. RM44 was monomorphic between the parents Zhe733 and R312 and thus could not be used in previous linkage mapping study [14, 34]. The polymorphism information content (PIC) of RM44 (0.28) was much lower than the average (0.45) over the other 70 markers within group 2. Group 2 had 25 resistant accessions accounting for 67.6% of the resistant accessions in this diverse population, including resistant Zhe733 with the 101 bp allele of RM44. However in group 2, 20 resistant accessions had the 101 bp allele, 25 moderately resistant accessions had the 101 bp allele, and 40 susceptible accessions had the 101 bp allele as well. Similarly, RM284 close to* qSH-8* was not associated with straighthead. These results suggest that the low levels of polymorphism within group 2 could decrease the power of QTL identification during association mapping.

On the other hand, the power of structure-based association analysis to detect the effects of individual genes is limited when population structure is found to explain too much of the variation [35]. By estimating the variation of allele frequencies (*θ*) at each locus among the groups in the diverse collection [36], we found that *θ* of locus RM284 near* qSH-8* was higher (0.54) than the average of other 70 markers (0.44), which indicated that the allele frequencies of RM284 were different from one group to another. In other words, the distributing pattern of RM284 alleles was in accordance with population structure. The accordance also can compromise the potential of marker to associate with gene(s)/QTL when population structure is taken in account in a model of association mapping. The similar result is also observed in another study [37]. In these cases, alternative populations for association mapping need to be evaluated for the candidate polymorphisms [37]. Additionally, an increase of marker coverage (i.e., high density SNP coverage) is a good option to increase the likelihood of polymorphic marker(s) and decrease the impact of population structure.

## Figures and Tables

**Table 1:** Identification of marker associated with straighthead responding to MSMA. Marker-straighthead associations detected with PCA model at *P* and *q*FDR value < 0.0001 and their position (cM) on chromosome (Chr) derived from 71 markers and 380 accessions in a diverse rice collection.

Maker	Chr	*P* value	*q*FDR
RM475^*∗*^	2	6.80 × 10^−8^	6.65 × 10^−7^
RM263	2	1.58 × 10^−5^	6.18 × 10^−5^
RM169	5	2.95 × 10^−6^	1.45 × 10^−5^
RM224	11	1.77 × 10^−7^	1.15 × 10^−6^
RM19	12	1.28 × 10^−8^	2.51 × 10^−7^

^*∗*^RM475 was also associated with straighthead resistance in linkage mapping of the F_2_ population derived from *Zhe733*/*R312 *[14].
